# Use of AFLP and RAPD molecular genetic markers and cytogenetic analysis to explore relationships among taxa of the Patagonian *Bromus setifolius* complex

**DOI:** 10.1590/S1415-47572009005000029

**Published:** 2009-06-01

**Authors:** Ana M. García, Gustavo E. Schrauf, Graciela González, Lidia Poggio, Carlos A. Naranjo, Marck P. Dupal, Germán C. Spangenberg, John W. Forster

**Affiliations:** 1 Facultad de Ciencias Exactas y Naturales, Universidad de Buenos Aires, Buenos AiresArgentina; 2 Facultad de Agronomía, Universidad de Buenos Aires, Buenos AiresArgentina; 3 Instituto Fitotécnico de Santa Catalina, LlavallolArgentina; 4 Primary Industries Research Victoria, Victorian AgriBiosciences Centre, Bundoora, VictoriaAustralia

**Keywords:** AFLP, Argentina, *Bromus setifolius complex*, cytogenetics, Patagonia, RAPD

## Abstract

*Bromus setifolius* var. *pictus* (Hook) Skottsb., *B. setifolius* var. *setifolius* Presl. and *B.setifolius* var. *brevifolius* Ness are three native Patagonian taxa in the section *Pnigma* Dumort of the genus *Bromus* L. AFLP and RAPD analysis, in conjunction with genetic distance measurements and statistical techniques, revealed variation within this group and indicated that *B. setifolius* var. *brevifolius* was closely related to *B. setifolius* var. *pictus*, with both taxa being more distantly related to *B. setifolius* var. *setifolius*. Cytogenetic analysis confirmed the chromosomal number of *B. setifolius* var. *pictus* (2*n* = 70) and *B. setifolius* var. *setifolius* (2*n* = 28) and showed for the first time that *B. setifolius* var. *brevifolius* had 2*n* = 70. The combination of molecular genetic and cytogenetic evidence supported a species status for two of the three taxa and suggested hypotheses for the evolutionary origin of these complex taxa. Species status was also indicated for *B. setifolius* var. *setifolius*. Based on these findings, we suggest that *B. setifolius* var. *pictus* be referred to as *B. pictus* Hook var. *pictus*, and *B. setifolius* var *brevifolius* as *B. pictus* Hook var *brevifolius*. The correlation between AFLP diversity and variation in ecological parameters suggested that this marker system could be used to assess breeding progress and to monitor the domestication of Patagonian *Bromus* species for agronomic use.

## Introduction

The genus *Bromus* L. contains approximately 150 species grouped into six taxonomic sections that are distributed throughout temperate regions of the world (Clayton and Renvoize, 1986). The section *Pnigma* includes about 60 species that are disseminated across Eurasia and North and South America (Smith, 1970). In Patagonia of South America, the section *Pnigma* is represented by *B. setifolius* Presl., which has been considered as a complex taxon because of its extensive morphological variation (Camara Hernandez, 1978; Matthei, 1986; Gutierrez and Pensiero, 1998). Three varieties of this species are recognized, namely, *B. setifolius* var. *setifolius*, *B. setifolius* var. *pictus* (Hook. F.) Skottsberg and *B. setifolius* var. *brevifolius* Ness.

The semi-desert region of Patagonia is a large environmental niche that has been subjected to substantial degradation (Aguiar and Sala, 1998). Bare soils represent ~60% of the total area, and a recent history of over-grazing has accelerated the process of desertification. Members of the *B. setifolius* complex could play an important role in the cessation and reversal of this environmental degradation (Aguiar and Sala, 1997). This is particularly true of *B. setifolius* var. *pictus*, which shows a favorable combination of drought resistance, high recruitment capacity and forage nutritive value. *Bromus setifolius* var. *pictus* and *B. setifolius* var. *setifolius* are sympatric inhabitants of the Patagonian semi-desert, whereas *B. setifolius* var. *brevifolius* is found in wetter grass steppe environments of the sub-Andean region (Naranjo *et al.*, 1990; Gutierrez and Pensiero, 1998).

Naranjo *et al.* (1990) performed a biosystematic study of the two sympatric taxa *B. setifolius* var. *setifolius* and *B. setifolius* var. *pictus*. Numerical taxonomy based on cluster analysis and principal components analysis (PCA) of 35 exomorphological characters detected two phenetically distinct groups that corresponded to *B. setifolius* var. *setifolius* and *B. setifolius* var. *pictus*. Cytological examination of mitosis demonstrated that *B. setifolius* var. *setifolius* plants had a chromosomal composition of 2n = 4x = 28, whereas *B. setifolius* var. *pictus* plants had a chromosomal composition of 2n = 10x = 70. Other cytogenetic differences between the two taxa included the karyotypic formula, type of satellite chromosome, basic genome length, asymmetry, C-banding patterns, and meiotic chromosomal behavior.

Meiotic chromosomal pairing in *B. setifolius* var*. setifolius* indicated the formation of 14 bivalents, typical of an allotetraploid. In contrast, chromosomal pairing in *B. setifolius* var. *pictus* suggested segmental allodecaploid behavior, with the formation of 35 bivalents being the most frequent configuration. With regard to the breeding systems, cleistogamous and chasmogamous flowers were found in *B. setifolius* var. *setifolius* whereas only cleistogamous flowers were observed in *B.setifolius* var. *pictus*; morphological and cytological analyses revealed no hybrid individuals. The stable morphological features of both taxa, which paralleled the karyotypic differences between them, suggested that they should be recognized as separate species (Naranjo *et al.*, 1990). Although several cytogenetic studies have been reported for taxa of the *B. setifolius* complex (Covas and Schnack, 1946; Stebbins, 1947; Covas and Hunziker, 1954; Naranjo *et al.*, 1990), the chromosome number of *B. setifolius* var. *brevifolius* has not as yet been determined.

Robust delineation among taxa within *B. setifolius* is problematic (Gutierrez and Pensiero, 1998). The morphological traits that distinguish these taxa are not randomly selected and may consequently bias estimates of genetic distance among groups (Lewontin, 1974). In addition, the discontinuity of morphological characteristics is not always observed. Although the potential influence of environmental selection on adaptive traits is more readily understood for phenotypically-apparent traits than for cryptic markers such as DNA-based polymorphisms, genetic polymorphisms may nevertheless provide complementary data for detailed analysis of closely related genotypes.

Molecular genetic methods have been applied to plant biosystematics with considerable success (Doebley, 1992; Soltis and Soltis, 1998). Random amplified polymorphic DNA (RAPD; Williams *et al.*, 1990) and amplified fragment length polymorphism (AFLP; Vos *et al.*, 1995) are two PCR-based techniques that have been successfully applied to the identification and estimation of molecular genetic diversity in various species (Newbury and Ford-Lloyd, 1993; Paran *et al.*, 1998; Kolliker *et al.*, 2001; Massa *et al.*, 2001; Puecher *et al.*, 2001). These techniques provide major advantages compared to other molecular marker methods such as restriction fragment length polymorphism (RFLP) because of their reduced requirement for large quantities of genomic DNA, their ability to amplify DNA from preserved tissues, and the smaller amount of time, labor and cost involved. In addition, unlike the PCR-based simple sequence repeat (SSR) and single nucleotide polymorphism (SNP) techniques, no prior sequence knowledge is required, and both RAPD and AFLP generate multiple locus information in a single assay (Wolfe and Liston, 1998). However, problems with reproducibility, particularly for RAPD, and the generation of dominant markers that are not locus-specific, limit the applicability of these methods, especially for genetic mapping studies compared to simple DNA profiling.

The aim of this study was to use molecular genetic and cytological approaches to examine the relationships among taxa of the *B. setifolius* complex. The results described here should be useful in elucidating the evolutionary processes that led to the origin of these taxa and should facilitate the domestication of forage grass species with potential agronomic value.

**Figure 1:**
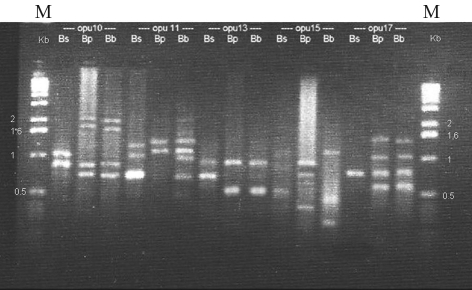
RAPD electrophoretic profiles from pooled samples of *B.  setifolius* var. *setifolius (Bs), B. setifolius* var. *pictus (Bp)* and *B. setifolius*var. *brevifolius (Bb)* based on five primers (opu10: 5’-ACCTCGGCAC- 3’, opu11: 5’-AGACCCAGAG-3’, opu13: 5’-GGCTGGTTCC-3’, opu15: 5’-ACGGGCCAGT-3’, opu17: 5’-ACCTGGGGAG-3’). Molecular size markers are indicated (M = 1 kb DNA ladder).

**Figure 2:**
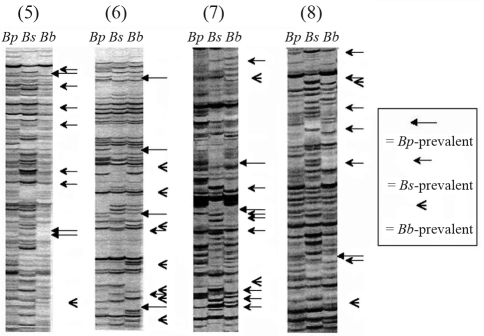
AFLP electrophoretic profiles from pooled samples of *B.  setifolius* var. *pictus (Bp)*, *B. setifolius* var. *setifolius (Bs)* and *B. setifolius* var. *brevifolius (Bb)* based on EcoRI/MseI primer combinations (5), (6), (7) and (8). Arrows indicate species-prevalent markers.

**Figure 3:**
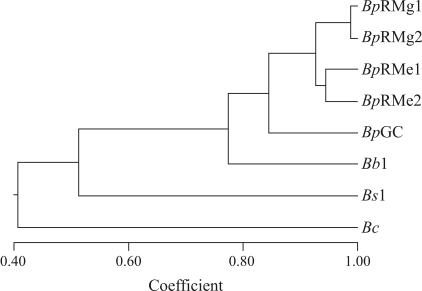
UPGMA dendrogram for *B. setifolius* var. *setifolius (Bs1)*, *B. setifolius* var. *brevifolius (Bb1)* and *B. setifolius* var. *pictus (Bp)* accessions and the *B. catharticus* control (*Bc*).  The Jaccard similarity coefficients of AFLP marker data were used to construct the dendrogram.

**Figure 4:**
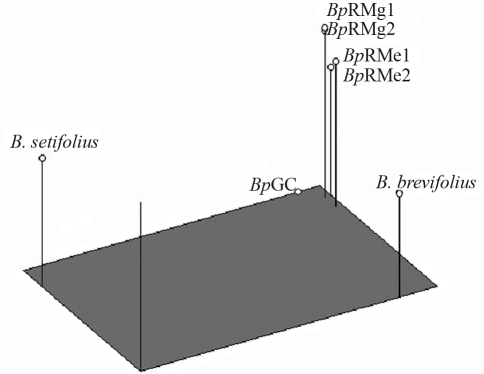
PCOA 3-D plot for *B. setifolius* var. *setifolius (Bs1)*, *B. setifolius* var. *brevifolius (Bb1)* and *B. setifolius* var. *pictus (Bp)* accessions and the *B. catharticus* control (*Bc*), based on the Jaccard similarity coefficients for AFLP marker data.

**Figure 5:**
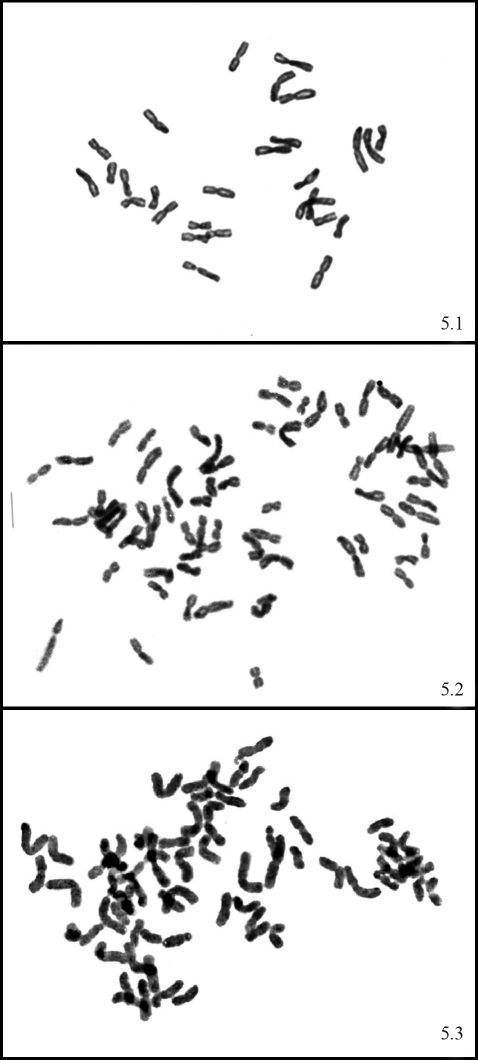
Photomicrographs of root tip metaphase chromosomes. 5.1 -*Bromus setifolius* var. *setifolius* (2n = 28), 5.2 - *Bromus setifolius* var. *pictus* (2n = 70), 5.3 - *Bromus setifolius* var. *brevifolius* (2n = 70).  Scale bar = 10 μm.

## Materials and Methods

### Plant material

Samples from different accessions of the section *Pnigma* were obtained from Patagonia, in Argentina (Table 1). The morphological variation of dried tussocks was used to classify each accession. Seeds obtained from different tussocks were planted in pots to produce plantlets for analysis. RAPD and AFLP analyses were done on pooled samples from 20 plantlets of *B. setifolius* var. *pictus* (*Bp*1), *B. setifolius* var. *brevifolius* (*Bb*1) and *B.setifolius* var. *setifolius* (*Bs*1). Five accessions of *B. setifolius* var. *pictus* were included in the AFLP analysis. One was native to a region close to the Atlantic coast known as Gobernador Costa, and is denoted here as *Bp*GC. The other four accessions were native to the Rio Mayo region in the Patagonian shrub steppe located close to the Andes: two of the Rio Mayo accessions were from areas grazed predominantly by sheep (denoted here as *Bp*RMg1 and *Bp*RMg2), and two were from areas excluded from the grazing by large herbivores, sheep and guanacos (denoted as *Bp*RMe1 and *Bp*RMe2). A pooled genotypic sample of *B. catharticus* Vahl. subsp. *stamineus* (Desv.) Massa (2*n* = 42) from section *Ceratochloa* of the genus *Bromus* was obtained from humid microenvironments of Patagonia and used as a control (outgroup).

### RAPD and AFLP assays

Young leaves from 20 plantlets of each accession were combined, snap frozen in liquid nitrogen and lyophilized for 48 h. Subsequently, aluminum oxide crystals were added and the samples were ground to a fine powder with a pre-cooled pestle and mortar, prior to DNA extraction by the method of Saghai-Maroof *et al.* (1984).

The amplification reactions for RAPD analysis was done using different decamer oligonucleotide primers (Operon Technologies, Almeda, USA) in a DNA thermal cycler (Perkin Elmer). After an initial denaturation step at 94 °C, 42 cycles of 45 s at 94 °C (denaturation), 60 s at 55 °C (annealing), and 90 s at 72 °C (extension) were done prior to a final extension of 5 min at 72 °C and subsequent cooling to 4 °C. The amplified products were visualized by staining with ethidium bromide (0.5 μg/mL) after electrophoresis at constant voltage (60 V) in 0.9% (w/v) agarose gels in TAE (40 mM Tris-acetate plus 1 mM EDTA) buffer. Seven microliters of the reaction mixture was added to 3 μL of 1 x gel loading buffer (Maniatis *et al.*, 1982).

AFLP reactions were done as described by Vos *et al.* (1995) with appropriate modifications. For digestion, 2.0-3.0 μg of the target DNA, 1.25 μL of *EcoR*I enzyme (12.5 units, 10 U/μL) and 1.25 μL of *Mse*I enzyme (12.5 units, 10 U/μL) were used in a reaction volume of 50 μL. Adaptors specific for each restriction enzyme terminus were ligated to the digested DNA fragments in a final volume of 60 μL followed by incubation at 37 °C for 5-6 h. Primary amplification by PCR was done using a +0/+0 primer combination and 30 cycles of 94 °C for 30 s, 56 °C for 60 s and 72 °C for 60 s. Diluted primary amplification product was selectively amplified by using 1 μL aliquots of the selected primer specific for the frequent-cutter restriction enzyme *Mse*I (30 ng/μL) and the rare-cutter restriction enzyme (*EcoR*I) (5 ng/μL). The primer for *EcoR*l was end-labelled with γ^33^P-ATP to allow autoradiographic detection of AFLP products. The primer combinations used for *Eco*RI/*Mse*I were: (1) AAA/GGGT, (2) AAG/GGGT, (3) AAG/CCA, (4) AAC/CCA, (5) AAC/GGG, (6) AAC/AAA, (7) AAA/CCA and (8) AAG/CAA. Selective amplification was done using the following touchdown PCR conditions: cycle 1: 94 °C for 30 s, 65 °C for 30 s and 72 °C for 60 s; cycles 2-13: 94 °C for 30 s, 65 °C for 30 s, with a 0.7 °C decrease in each cycle, and 72 °C for 60 s; cycles 14-36: 94 °C for 30 s, 56 °C for 30 s and 72 °C for 60 s. After amplification, the sample was mixed with one volume of sequencing loading buffer, heated to 95 °C for 5 min and briefly quenched on ice before the loading onto the gel. Samples were run on a 6% (w/v) denaturing polyacrylamide gel (19:1 acrylamide:bis, w/w) in 7.6 M urea and 1x TBE buffer. Electrophoresis was done at constant power (110 W) for ~2 h, after which the gel was dried at 80 °C for 40 min and analyzed by overnight exposure to a storage phosphor screen for phosphorimaging analysis (Molecular Dynamics).

### Analysis of molecular marker data

RAPD and AFLP bands were treated as dominant genetic markers and scored as polymorphic features if present in some but not all accessions, and as monomorphic features when the band was present in all of the accessions evaluated. To avoid underestimating the genetic similarity, the monomorphic and polymorphic bands were both included in the analysis. For RAPD data, genetic distance was estimated using the index of Balakrishnan and Sanghvi (1968). Matrices generated by scoring the AFLP bands were used as input data for the application NTSYS-pc version 2.01 (Rohlf, 1988). The Jaccard similarity index was applied using the SAHN program of NTSYS, and cluster analysis by UPGMA (unweighted pair-group method with arithmetic averages) was used done to obtain dendrograms. The data was also used for multidimensional principal coordinate analysis (PCOA).

### Cytogenetic analysis

Chromosomal number and morphology were determined in mitotic metaphases of 20 plants from each accession (*B. setifolius* var. *setifolius*, *B. setifolius* var. *brevifolius* and *B. setifolius* var. *pictus*; *Bs*1, *Bb*1 and *Bp*1, respectively). Seeds from accessions *Bs*1, *Bb*1 and *Bp*1 were placed on wet filter paper in Petri dishes. Root tips were incubated in ice water for 36 h, fixed in ethanol-glacial acetic acid (3:1, v/v) for 2 days at 4 °C, and then transferred to 70% ethanol and refrigerated until analyzed. Squashed preparations were prepared in 2% hematoxylin in acetic acid and examined with a Zeiss Axiophot microscope. Photographs were taken with Kodak T-Max-100 film.

## Results

### RAPD analysis

Of the 20 decamer RAPD primers that were tested for PCR amplification, 11 yielded clear, reproducible amplification profiles with the three pooled accessions. Thirty-three of 41 clearly distinguished bands were polymorphic (Figure 1). Calculation of the genetic distances showed that *B. setifolius* var. *pictus* and *B. setifolius* var. *brevifolius* were closer to each other than either was to *B. setifolius* var. *setifolius* (Table 2).

### AFLP analysis

The amplification profiles obtained with different AFLP primer combinations in the seven samples corresponding to different accessions were used to score 692 fragments. The number of bands in each accession differed among taxa, with *B setifolius* var. *setifolius* having a lower number of bands (439) than *B. setifolius* var. *brevifolus* (466) and *B. setifolius* var. *pictus* (an average of 471). Within *B. setifolius* var. *pictus*, accessions obtained from grazed areas had a lower number of bands (466) than accessions from non-grazed areas (an average of 477). Two hundred and seventy-seven bands were monomorphic among the taxa and 415 bands were polymorphic. Among the polymorphic bands, 153 were exclusive to a single taxon: *B. setifolius* var. *setifolius* had 100 exclusive bands, *B. setifolius* var. *brevifolius* 35 and *B. setifolius* var. *pictus* 18. Within *B. setifolius* var. *pictus*, 429 bands were monomorphic and 84 were polymorphic. A comparison of the amplification profiles among accessions in this taxon allowed the identification of accession-prevalent and species-prevalent markers (Figure 2).

Samples *Bp*RMg1 and *Bs*1 were from the same location and, in some cases, the same tussock included both species. In contrast, in the other species, individual tussocks contained only one species, but generally included plants with more than one genotype for the species. The genetic distances among the samples analyzed by AFLP were calculated by using the Jaccard similarity coefficient (Table 3). The degree of similarity among the five accessions of *B. setifolius* var. *pictus* was high (0.834-0.988). Figure 3 shows the dendrogram obtained by cluster analysis of the similarity indexes using the UPGMA method (Figure 3).

The pooled sample of *B. catharticus* genotypes was clearly separated from accessions of the section *Pnigma*, thus confirming that *B. catharticus* was an outgroup in this analysis. The three taxa within section *Pnigma* were grouped in a single large cluster. Within *B. setifolius* var*. pictus*, accessions from the same geographical area (Rio Mayo) were grouped in the same sub-cluster. Two additional sub-clusters were identified in accessions from this geographical location and differed in their grazing history.

Figure 4 shows a 3-dimensional plot of the AFLP data based on multidimensional PCOA. The first principal coordinate (depicted by the length of the rectangle in Figure 4) explained 51.58% of the total variation and separated *B. setifolius* var. *setifolius* from *B. setifolius* var. *pictus* and *B.setifolius* var. *brevifolius*. The second principal coordinate (depicted by the width of the rectangle) explained 21.52% of the variation and separated *B. setifolius* var. *brevifolius* sample from *B. setfolius* var. *pictus* and *B. setifolius* var. *setifolius*. The third principal coordinate (depicted by the height of the bars) explained 15.03% of the variation and discriminated between the populations of *B.setifolius* var. *pictus*. Each coordinate in multidimensional PCOA corresponded to a new variable associated with genetically-related interspecific differences identified by AFLP analysis, and indicated that the greatest genetic divergence was between the *B. setifolius* var. *brevifolius* and *B. setifolius* var. *setifolius* samples (Table 3).

### Cytogenetic analysis

Cytogenetic analysis revealed chromosomal numbers of 2n = 28 for *B. setifolius* var. *setifolius*, 2n = 70 for *B. setifolius* var. *pictus* and 2n = 70 for *B. setifolius* var. *brevifolius* (Figure 5).

## Discussion

### Molecular marker-based genetic diversity analysis

RAPD and AFLP analyses of taxa in the genus *Bromus* (section *Pnigma*) yielded similar results. Although difficulties associated with the reproducibility of RAPD limit the applicability of this technique (Perez *et al.*, 1998), RAPD may nevertheless be useful for preliminary assessments of intraspecific, interspecific and intergeneric genetic variation in grasses (Wang *et al.*, 2001; Garcia *et al.*, 2004). In contrast, the greater accuracy and information content of AFLP make this technique more useful for detailed investigations such as in molecular breeding.

The variation in the number of bands among accessions was relatively small when compared to the large differences in chromosome numbers among taxa. Despite its lower chromosome number the tetraploid taxon *B. setifolius* var. *setifolius* had only 6.7% fewer bands than the decaploid taxon *B. setifolius* var. *pictus.* The higher content of genomic DNA in *B. setifolius* var. *setifolius* (1 Cx = 2.06 pg) compared to *B. setifolius* var. *pictus* (1 Cx = 1.63 pg) (Naranjo *et al.*, 1990) may compensate for the lower chromosome number and account for the complexity of its AFLP profile. Fay *et al.* (2005) reported that the number of AFLP bands tended to increase up to a DNA content of 1C = 8.43 pg.

*Bromus setifolius* var. *pictus* and *B. setifolius* var. *brevifolius* were more similar to each other than either was to *B. setifolius* var. *setifolius*. This observation is consistent with studies of morphological variation in which *B. setifolius* var. *pictus* and *B.setifolius* var. *brevifolius* showed a high degree of similarity and were difficult to distinguish taxonomically (Camara Hernandez, 1978). Although variation in the morphological traits did not permit a clear delineation between the taxa (Gutiérrez and Pensiero, 1998), both RAPD and AFLP analysis revealed bands that may be considered as taxon-specific discriminatory markers. This was particularly the case in the AFLP profiles. However, these results must be treated as preliminary until a larger number of accessions have been analyzed. AFLP analysis showed that very low frequencies in one taxon and very high frequencies in another may be readily misclassified as diagnostic bands because of the small sample sizes involved. Consequently, such features may be more accurately described as taxon-prevalent and require more intensive analysis.

Analysis of the genetic structure of plant populations requires the sampling of individual plants. In grasses of the genus *Bromus*, it is conventional to consider each tussock as an individual genotype, but it is also possible for individual tillers in a single tussock to be derived from different individuals of the same species, or even from different species. Wind damage on the Patagonian steppe produces high mortality during seedling recruitment (Aguiar and Sala, 1997). Consequenly, long-established tussocks may provide a ‘nurse effect' by protecting the seedlings against wind. The composite content of individual tussocks is a potentially important confounding factor in the study of genetic diversity and may also provide an explanation for some of the difficulties previously encountered in the taxonomic discrimination of related species. The use of individual inflorescences as the unit of study, rather than the entire tussock, provides a way to address this problem.

### Relationships between genetic variation and ecological parameters

In grass species there is not always a correlation between variation in morphological traits, geographical distribution, life history traits and biochemical or molecular genetic data. The lack of such correlations for *B. catharticus* was explained in terms of phenotypic plasticity (Puecher *et al.*, 2001). In the grass *Schizachyrium scoparium* (Carman and Briske, 1985) morphological differences were detected between populations that had been subjected to long-term grazing and those that had not been grazed, but isoenzyme variation was not related to grazing history. Schrauf and Naranjo (1988) attributed the observed differences in isozenzyme frequencies between grazed and non-grazed populations of *Pappophorum caespitosum* and *P. philippianum* to the random effects of genetic drift.

As shown here, accessions of *B. setifolius* var. *pictus* with different grazing histories were resolved into different sub-clusters based on AFLP data. In addition, non-grazed accessions had more AFLP bands than those that had been grazed. These results may have several explanations. Sheep, as the primary grazing animals in this study, may have a more selective grazing behavior than bison (Carman and Briske, 1985) and cows (Schrauf and Naranjo, 1988), as found for *Danthonia* species (Scott and Whalley, 1984). Alternatively, the wider range of markers analyzed by AFLP makes this system more capable of assessing a more representative sample of genomic variation than isoenzymes, thereby allowing finer discrimination among accessions.

### Cytogenetic analysis and evolutionary relationships

Kozuharov *et al.* (1981) showed that diploid, tetraploid and higher polyploid level chromosomal races within the annual *Bromus* species *B. secalinus* and *B. lanceolatus* were morphologically similar and that there was little justification for their taxonomic separation. In contrast, the perennial octoploid species *B. lacmonicus* and the diploid species *B. parilicus* are morphologically similar but show features that reflect their cytological differences and consequently justify their species status. Similarly, *B. setifolius* var. *setifolius* (2n = 28) and *B. setifolius* var. *pictus* (2n = 70) are perennial taxa with stable morphological differences that parallel their karyotypic divergence and support their classification as separate species.

Naranjo *et al.* (1990) proposed that *B. setifolius* var. *setifolius* (*sensu* Naranjo *et al.* (1990): *B. setifolius*) may be a progenitor taxon of the polyploid *B. setifolius* var *pictus* (*sensu* Naranjo *et al.* (1990): *B. pictus*) on their morphological similarity, karyotypic structure, growth pattern, breeding system and presence of reproductive isolation. Recent work using fluorescence *in situ* hybridization with total genomic DNA as a probe (GISH) has shown that *B. setifolius* var*. setifolius* or a closely related taxon is one of the progenitors of *B. setifolius* var. *pictus*, thus confirming the allopolyploid nature of this species (Garcia *et al.*, 2005).

This report is the first to describe the chromosomal number of *B. setifolius* var. *brevifolius* (2n = 70). As shown here, molecular genetic data indicated a close relationship between *B. setifolius* var. *brevifolius* and *B. setifolius* var. *pictus*, and AFLP-based PCOA suggested that *B. setifolius* var. *brevifolius* may be directly derived from *B. setifolius* var. *pictus*, rather than the second allopolyploid progenitor, in agreement with the morphophysiological reticular pattern (Garcia *et al.*, 2001). *Bromus setifolius* var. *brevifolius* has wide leaves and a fast initial growth, *B. setifolius* var. *setifolius* has short internodes and high drought resistance, and *B. setifolius* var. *pictus* has wide leaves, short internodes, fast initial growth and high drought resistance. The putative progenitor of *B. setifolius* var. *pictus* may have had a haploid chromosomal complement of n = 21, assuming that *B.setifolius* var. *pictus* has retained the genetic complement of both ancestral species (some chromosomal rearrangements may have occurred subsequent to polyploid formation). Additional studies are required to determine the identity of this progenitor.

### Conclusions and agronomic implications 

A taxonomic realignment of *Bromus* species within section *Ceratochloa* has been proposed based on morphological, cytological and molecular genetic data (Massa e*tal.*, 2004). The molecular genetic and cytogenetic data described here have clarified the relationships among taxa of the Patagonian *Bromus* section *Pnigma*. These data agree with the findings of Naranjo *et al.*. (1990). Taxonomically, *Bsetifolius* var. *setifolius* and *B. setifolius* var. *pictus* should be recognized as distinct species under the names *B. setifolius* Presl. and *B. pictus* Hook, respectively. The cytological and molecular genetic similarity between *B. setifolius* var. *pictus* and *B.setifolius* var. *brevifolius* suggests that these taxa be considered as varieties within the same species, *i.e.*, *B. pictus* var. *pictus* and *B. pictus* var. *brevifolius*, respectively.

*Bromus pictus* var. *pictus* is currently the most attractive option for a cultivated species to mediate the reversal of environmental degradation in Patagonia because of its combination of drought tolerance, high recruitment capacity and forage quality. Biodiversity conservation issues may also be addressed through domestication and improved breeding of *B. pictus* var. *pictus*, particularly since genetic erosion through sheep grazing has pushed species of the section *Pnigma* to the brink of extinction. The genetic conservation of *B. pictus* var. *pictus* will prove effective for the section as a whole, and the initial data described here for molecular marker variation will provide valuable information for marker-assisted breeding and future evolutionary studies.

## Figures and Tables

**Table 1:** Classification of the samples used for molecular genetic marker analysis based on the species and variety, geographical origin, sample code and type of analysis.

Species	Population	Code	Type of molecular marker analysis
*B. s.* var. *setifolius*	Río Mayo	*Bs*1	RAPD and AFLP
*B. s.* var. *brevifolius*	San Martín de los Andes	*Bb*1	RAPD and AFLP
*B. s.* var. *pictus*	Río Mayo	*Bp*1	RAPD
*B. s.* var. *pictus*	Río Mayo (non-grazed 1)	*Bp*RMe1	AFLP
*B. s.* var. *pictus*	Río Mayo (non-grazed 2)	*Bp*RMe2	AFLP
*B. s.* var. *pictus*	Río Mayo (grazed 1)	*Bp*RMg1	AFLP
*B. s.* var. *pictus*	Río Mayo (grazed 2)	*Bp*RMg2	AFLP
*B. s.* var. *pictus*	Gobernador Costa	*Bp*GC	AFLP
*B. catharticus*	Control (outgroup)	*Bc*	AFLP

**Table 2:** Genetic distances among accessions of *Bromus* in the section *Pnigma*, based on RAPD analysis of pooled samples. The distances were calculated as described by Balakrishnan and Sanghvi (1968).

	*B. s.* var. *pictus*	*B. s.* var. *brevifolius*	*B. s.* var. *setifolius*
*B. s.* var. *pictus(Bp1)*	0	-	-
*B. s.* var. *brevifolius (Bb1)*	37.9	0	-
*B. s.* var. *setifolius (Bs1)*	56.1	68.2	0

**Table 3:** Genetic distances among accessions of *Bromus* in the section *Pnigma*, based on AFLP analysis of pooled samples. The distances were calculated by using the Jaccard similarity coefficient.

Accession	*Bp*RMg1	*Bp*RMg2	*Bp*RMc1	*Bp*RMc2	*Bp*GC	*Bs*1	*Bb*1
*Bp*RMg1	1	-					
*Bp*RMg2	0.988	1					
*Bp*RMe1	0.924	0.927	1				
*Bp*RMe2	0.927	0.930	0.943	1			
*Bp*GC	0.834	0.841	0.863	0.839	1		
*Bs*1	0.529	0.530	0.506	0.521	0.502	1	
*Bb*1	0.783	0.786	0.777	0.805	0.719	0.485	1
